# Supplementary material for: Implementation of the WHO guideline on treatment of young infants with signs of possible serious bacterial infection when hospital referral is not feasible in rural Zaria, Nigeria: Challenges and solutions
Source: PLoS One. 2020 Mar 10;15(3):e0228718. doi: 10.1371/journal.pone.0228718 (PMC7064229; doi:10.1371/journal.pone.0228718)
Supplement: Attachment — Submitted filename: Response to Reviewers.docx [file pone.0228718.s002.docx]

January 12, 2020

Dear Editor,

We wish to thank you for considering our manuscript for publication in your journal. Please find below our response, point by point to the issues raised by the academic editor and reviewers.

| **Additional Editor Comments (if provided):** | | |
| --- | --- | --- |
| S/No. | Editor’s questions | Author responses |
| 1. | Given that the study was designed and presented as "implementation research, please specify if a formal implementation model or framework was used, and cite appropriate implementation science literature if relevant. | We adapted the RE-AIM framework for planning and evaluation of this study. This is explained in figure 1  **(**Harden SM, Smith ML, Ory MG, Smith-Ray RL, Estabrooks PA, Glasgow RE. RE-AIM in Clinical, Community, and Corporate Settings: Perspectives, Strategies, and Recommendations to Enhance Public Health Impact. *Front Public Health* 2018; **6**: 71.) |
| 2. | Was there a specific reason to select Zaria LGA as the study site? | A policy dialogue at the central level was held in Abuja, in which State and local government functionaries and other technical experts took part. It was decided that the national policy should be adapted to implement WHO PSBI guideline when referral is not feasible in two places in Nigeria, one in the North part of the country and one in the South to represent both diverse regions. In the North part of the country, Kaduna state was proposed and in South part, Oyo State was proposed. Zaria LGA is representative of a population where referral is not accepted by many families with sick young infants. We also held consultations with the local authorities and in consultation chose certain wards in Zaria LGA to implement this guideline. We have now described this in methods. |
| 3. | The study describes both development of the implementation, evaluation of outcomes, as well as some description of challenges and solutions within the implementation process – please state explicitly whether these were studied systematically or based on anecdotes, team meetings etc. For example, were specific questions asked of field workers periodically to understand barriers? This should be included in the methods. | a. The evaluation of outcomes was studied systematically. All young infants receiving treatment were assessed for these outcomes and documented on study case record forms. b. The documentation of challenges is based on team meetings that included field workers, health workers at the facilities, supervisors and health facility managers, field assessments of the health facilities, asking families during follow-up visits to understand the barriers and identify facilitators to implement this intervention. We have included this in methods. |
| 4. | The Methods section "Evaluation of existing PHCs, health workers and commodities" includes several results of those evaluations: those results are better suited for the results, particularly as additional results of that evaluation are described in the Results section under Challenges and Solutions e.g., general shortage of staff at PHCs. Alternatively, if you prefer to keep some of this is the methods section, state explicitly that this was pre-implementation evaluation to inform the intervention implementation, and tie it into the paragraph that immediately follows (establishment of TSU, and how this evaluation informed what the TSU needed etc.). | We chose to keep some of the descriptions in the methods and presented it as pre-implementation evaluation to inform the intervention for the implementation of the guideline and link it with the TSU work. This is described now in the manuscript |
| 5. | Please limit the use of abbreviations and acronyms where possible e.g., TBA acronym is not necessary. Provide  reference for Integrated Community Case Management (iCCM) (acronym not necessary as it is not used again) | a. The use of abbreviations and acronyms have been limited to where they are necessary. b. Reference for iCCM has been provided. |
| 6. | In the discussion, it is stated that “No adverse event such as an injection abscess was reported.” Were these data  collected systematically or is this just anecdotal? | The data on adverse events was collected systematically during the study. Each newborn was evaluated for adverse event during follow visits and documented in the case record forms. |
| 7. | Consider adding some discussion on how the feasibility and implementation experience compares with similar  interventions in other LMIC. | We have compared our data with other implementation experience from a few LMIC countries. It has been added as suggested by the reviewer. |
| **Review Comments to the Author:**  **Reviewer #1: MAJOR COMMENTS:** | | |
| S/No. | Reviewer’s questions | Author responses |
| 1. | Some of the work described to implement this guideline in rural northern Nigeria included working with the national MOH and NGOs. I did not find a description of how more local public health and child health experts were engaged. | A policy dialogue was held at the central level, in which federal, representatives of the state and local governments, academics, researchers, non-governmental organizations, donors and technical experts took part. It was decided that the national policy should be adapted to implement WHO PSBI guideline when referral is not feasible in two places in Nigeria, one in the North and one in the South of the country to represent both very diverse regions. In the North Nigeria Kaduna state was proposed and in South of Nigeria Oyo State was proposed. The Kaduna State and Zaria LGA personnel were represented in that policy dialogue process. After that central level discussions, meetings and discussions were held with the health and other officials at Kaduna state and at the Zaria LGA level to get their buy-in and to identify appropriate locations for this implementation research. Discussions were also held with the local Amir (traditional ruler) and area counselors/community leaders to get their approval. We wanted the study population to be representative of where referral was not accepted by most families with sick young infants. Thus after a broad consultation with Zaria local authorities and communities, we chose these study wards to implement this guideline. |
| 2. | Additional information would be valuable about how the specific communities within Zaria were chosen for this  implementation project. | Zaria LGA is made of up of 11 political wards. These two political wards were chosen as they are among the most rural of these political wards and with limited accessibility to referral centers. More details of discussions and consultative process with various stakeholders have been given under responses to question number 1 by the reviewer 1 and question 2 by the editor. |
| 3. | More definitions are needed for the data elements that were captured and the way in which data were collected.  This is particularly important for adherence to treatment, other adverse outcomes, etc. | These definitions are provided in table 2. For adherence to treatment, systematic record was kept for the number of doses of injectable gentamicin and oral amoxicillin given to a sick young infants. It has been further clarified in the methods. |
| 4. | The description of the study population is vague. To describe the impact of this intervention, it would be very valuable to have a more precise way to define and identify both patients that were in the population cohort as well as children identified as those members of the cohort who were identified as having PSBI. Was this a birth cohort in which babies were followed to 2 months of age or event (which ever came first)? How were members of the cohort identified (or numbers of members estimated) during the study period? Did all of this occur via registration of pregnant women by CORPs staff? Can you estimate the percentage of live births in the region that were captured by this strategy? | CORPs (community volunteers who were paid a small stipend) were identified and trained in the catchment area communities. They identified and registered pregnancies in their respective areas. They visited home and also counseled mothers on how to identify the sick young infants and where to seek care. The newborns were followed for up to 2 months of age. The study population were babies who developed signs of PSBI whose families either self-present to the Primary health Care facilities or were identified in their homes by the CORPs and were brought to the Primary Health facilities for assessment by the health facility workers. We had mentioned in the beginning of the results section that we might have missed 25% of the births in the study area. |
| 5. | Ascertainment of the babies that experienced PSBI is subject to bias, particularly for the families in the most remote areas under study. Can the authors provide us some data on the proportion of live births who had the anticipated home visits (days 1, 3,7) by CORP workers? | We have provided the proportion newborns visited on days 1, 3, and 7. This is reflected in the results section. |
| 6. | The authors seem to have retained infants identified with PSBI from regions outside of the study area in the group on which they report outcomes. These babies should be removed. | The focus of the study was management of young infants with signs of PSBI when referral for hospital care is not possible. The inclusion of young infants with signs of PSBI was not restricted to communities within the study area. If families outside the study communities decided to access health services for their sick infant at any health facility located within the study area and has signs of PSBI, the families of this infant were counseled for referral for hospital care. Families who could not take their sick infant to the referral health facility, they were treated at the primary health care facility according to the guidelines. Thus, included into the study. |
| 7. | The authors do an excellent job describing the social context in which they were working. However, it would be valuable to provide a bit more information about the economic context and penetration of other health initiatives. What were the professions of most people in these communities (e.g., traders, subsistence farming)? What was the coverage of health interventions such as basic vaccination? | The main profession of the people in these communities is subsistence farming. The study was not designed to evaluate the economic status of the people but most people are poor. |
| 8. | The authors provide a detailed description of the micro-economics of purchase and replenishment of antibiotics in the primary health centers as well as the fact that care provided in those sites was free. I am confused about what costs a family would have to bear to bring their child to a PHC, get antibiotics if prescribed, fund transport to a hospital if referred, and pay for hospital-based care if it is recommended. These economic barriers to guideline adherence should be described succinctly since this novel program that provides an alternate to hospital referral may be more attractive to families simply based on cost. | Families of infants with signs of PSBI identified by health workers at the first level health facility (primary health care) were first of all referred to the hospital for inpatient care. Unfortunately, many families could not take their infant to the referral center for various reasons. They were then offered simplified treatment with injectable and oral antibiotics on an outpatient basis according to the WHO PSBI guideline as adopted by the country.  The cost to bring the child to the PHC was only transport costs if they had to hire something to get there. The medicines at the PHC were provided free of cost.  However, if the family accepted referral to a hospital it would involve transport costs, hospital stay costs and cost of medicines along with lost wages.  One of the reasons for not accepting referral to the hospital may be financial, but in this locality there are also cultural and social issues that may prevent families in accepting inpatient care. |
| 9. | Some of the information provided in both the methods and results might be more effectively and efficiently communicated with use of figures and/or tables. Examples include 1) phases of implementation (figure), | A figure (figure 1) summarizing the phases of implementation and activities has been provided. |
| 10. | It seems as though there were multiple types of trainings developed and delivered. However, this is not clearly described. Please clarify and include who did the trainings, how the adequacy of training was assessed, and provide materials in supplement if possible. | There were two types of trainings carried.  I. Training on assessment, classification and management of young infants with PSBI signs when referral is not feasible using the young infant component of IMCI training package (Ref. 12 and 21). The training was in two stages. Initially, Master Trainers from all the study sites were trained by WHO facilitators for three days. Subsequently, the Master trainers provided onsite training to the Nurses and CHEWs in Zaria.  II. The second was an onsite 6-day training on home visits for care of the newborn and identifying sick young infants for CHEWs and CORPs conducted by the Master trainers supported by the study coordinators using the WHO training manual for care of the newborn at home (ref. 22). For CORPS training government approved training manual in Nigeria was also used. |
|  | **OTHER COMMENTS:** |  |
| 1. | The title does not contain several important pieces of information – that this is a pediatric study population and that a major part of the work described is about the development of a context-specific implementation plan. It may also be good to indicate in the title that this is about the implementation in a rural setting, since others may search to find reports of how to overcome the unique challenges associated implementation of health interventions where formal healthcare facilities are sparse. | Thank you for this comment. We have added the pediatric age and rural in the title. It now reads “Implementation of the WHO guideline on treatment of young infants with signs of possible serious bacterial infection when hospital referral is not feasible in rural Zaria, Nigeria: Challenges and solutions”. |
| 2. | The authors could use some assistance with correcting basic errors of syntax, which would improve the capacity of readers to comprehend some aspects of their work. Also need to standardize their approach to introduction and use of abbreviations. | We have tried to correct the syntax errors. |
| 3. | Several phrases are unclear to me: “patent medicine vendor”, “seed drugs” | Further explanations have been provided for these phrases. |
| 4. | I had a hard time keeping straight the various types of healthcare workers (CHEWs, CORPs, CHO, etc.). It might be good to provide a glossary or table that pulls together all of these abbreviations, definitions, setting in which they practice, and the scope of their practice. | A panel has been included to provide these definitions and scope of work for the various health workers. |
| 5. | What tool was used to “assess the status of health facilities” to determine capacity of existing resources to implement this guideline? Is this something that could be provided as part of supplementary materials? | We used the modified version of “Equipment and supply checklist” from the WHO health facility survey (Ref: World Health Organization. Health facility survey: tool to evaluate the quality of care delivered to sick children attending outpatient facilities. World Health Organization. Geneva 2003. <http://www.who.int/iris/handle/10665/42643> |
| **Review Comments to the Author:**  **Reviewer #2:** | | |
| 1. | The authors should include a detailed description of how training to lay people for them to administer IM injections to infants at appropriate dosages was done. This should include needle stick safety and disposal. | There seems to be some misunderstanding. The injections were only provided by either nurses working in the health centers, who receive four years formal training in accredited schools of nursing or CHEWs who receive two (junior CHEW) /three years (senior CHEW) training in accredited schools of health technology. They are certified by Nigeria health authorities to administer intramuscular injections.  The lay workers known as CORPs work in the communities and did not provide any injectable treatment. The CORPs were trained to only recognize signs of PSBI in the newborn and advise families to take their newborns to the health facilities. They were trained using government approved training manual for CORPs in Nigeria. |
| 2. | How rigorous was the adverse event recording documentation after IM injections? | All newborns receiving treatment were assessed on each visit to the health facility, which was daily, by the trained health facility workers and documented on the Case Record Forms for the study. We have clarified this in the manuscript. |
| 3. | The authors should include under "limitations" why they did not take blood cultures to validate the claim for bacterial infections in this population. Antimicrobial sensitivity would have guided in future antibiotic use and this could have provided baseline data | In Nigeria the primary health care facilities do not have any facilities for microbiology. As mentioned in the introduction, WHO categorizes these sick young infants as “possible serious bacterial infection (PSBI)” and recommends a syndromic approach without taking blood samples for cultures in the IMCI protocol. We did not have resources to provide good quality microbiology services in this rural area. |
| 4. | Figure 1 seems to suggest seasonal variation of infection. What are the RSV rates in this population? Viral infection usually precedes bacterial infections. | It is potentially possible. We do not have any local data from Zaria. Other published data shows that young infants presenting with PSBI signs can have only viral including RSV, only bacterial and mixed viral and bacterial etiology. (Saha SK, Schrag SJ, El Arifeen S, Mullany LC, Shahidul Islam M, Shang N, et al. Causes and incidence of community-acquired serious infections among young children in south Asia (ANISA): an observational cohort study. Lancet 2018; 392: 145 – 159.) |
|  |  |  |

Thank you, very much and best regards,

Wammanda Daniel Robinson

Department of Paediatrics, ABUTH, Zaria, Nigeria.
